# Meta-Analysis on the Efficacy and Safety of Traditional Chinese Medicine as Adjuvant Therapy for Refractory Androgenetic Alopecia

**DOI:** 10.1155/2019/9274148

**Published:** 2019-10-31

**Authors:** Qiang You, Lan Li, Xiao Ma, Tian Gao, Suqin Xiong, Yufen Yan, Hao Fang, Fengqing Li, Hongping Chen, Youping Liu

**Affiliations:** ^1^Department of Pharmacy, Chengdu University of Traditional Chinese Medicine, Chengdu, China; ^2^The Affiliated Hospital, Southwest Medical University, Luzhou, China; ^3^Department of Nursing, Southwest Medical University, Luzhou, China; ^4^The Affiliated Hospital, Chengdu University of Traditional Chinese Medicine, Chengdu, China

## Abstract

**Objective:**

Traditional Chinese medicine (TCM) therapies have been widely used for the treatment of androgenetic alopecia (AGA) for thousands of years. We conducted a meta-analysis to evaluate the curative efficacy and safety of TCM for treating AGA.

**Methods:**

Randomized controlled trials (RCTs) of TCM for the treatment of AGA through March 2019 were systematically identified in 4 English databases, namely, PubMed, Cochrane Library, EMBASE, and Web of Science, and 4 Chinese databases, namely, Sino-Med, China National Knowledge Infrastructure (CNKI), China Science and Technology Journal Database (VIP), and WanFang. Quality assessment and data analysis were performed by Review Manager 5.3.5, and Stata 15.1 was used to cope with publication bias.

**Results:**

30 RCTs involving 2615 patients were randomly divided into a TCM group and a conventional medicine (CM) group. The results showed that the total efficacy rate (TER) of the TCM group was significantly higher than that of the control group (OR = 3.34, 95% CI = 2.75–4.05, *P* < 0.00001). The total symptom score (TSS) of the TCM group was markedly reduced when compared with the CM group (SMD = −0.86; 95% CI = −1.19, −0.53; *P* < 0.00001). The microelement levels (Fe^2+^, Zn^2+^, and Cu^2+^) in hair were significantly improved when complemented with TCM therapy. In addition, no significant differences were observed between the two groups in terms of adverse events (OR = 0.55, 95% CI = 0.29–1.05, *P*=0.07).

**Conclusions:**

In view of the effectiveness and safety of TCM, the present meta-analysis suggests that TCM could be recommended as an effective and safe adjuvant therapy for the treatment of AGA by improving the TER, symptoms, serum testosterone levels, and microelement levels. However, long-term and higher-quality RCTs are needed to overcome the limitations of the selected studies and more precisely interrogate the efficacy and safety of TCM.

## 1. Introduction

Androgenetic alopecia, or male-pattern baldness, is one of the most common types of hair loss in both men and women, affecting approximately 0.2–2% of the world's population [1, 2]. Even if medically regarded as a relatively mild skin disease, alopecia leads to significant negative effects on the quality of life because of the importance of hair in people's psyche [2]. It was reported that at the age of fifty, the prevalence of male AGA was approximately 50% [3] and increased with age [4]. In China alone, hair loss affects more than 100 million men, and the aging of the rapidly growing global population is likely to aggravate this situation [5].

Modern medical research suggests that AGA is characterized by an autosomal dominant polygenic inheritance of alopecia and is highly associated with androgen-dependent miniaturization of scalp hair follicles and that dihydrotestosterone disorder is an important pathogenic factor [6]. Although much research has been devoted to antialopecia drugs, only two drugs, finasteride and minoxidil, have been approved by the FDA. Finasteride, a specific inhibitor of 5α-reductase, shows high irreversible binding affinity to the enzyme and inhibits the conversion of testosterone into highly active dihydrotestosterone, which provides an effective target for AGA treatment. However, three percent of patients experience sexual dysfunction [7]. These symptoms include decreased libido, diminished sexual function (impotence), decreased ejaculation volume, and ejaculation disorder [8]. Generally, minoxidil is an established therapy for AGA in both men [9, 10] and women [11]. Although the exact mechanism is unclear, contact dermatitis [12], skin irritation [13], dizziness, and tachycardia [14] have been reported. In addition, once the medication is discontinued, hair loss persists. Specifically, the efficacy and safety of the two drugs are unsatisfactory. Thus, research into effective treatments that can enhance the efficacy of finasteride and minoxidil is of great social and clinical significance.

Currently, an increasing number of AGA patients in East Asia have resorted to seeking effective and safe complementary therapies for AGA from traditional Chinese medicine (TCM). Traditional Chinese herbal formulas (CHFs) with fixed ingredients, recorded in the ancient Chinese classic medical books, have shown striking effects in the treatment of some chronic diseases such as AGA, chronic hepatitis, chronic gastritis, cerebrovascular diseases, and diabetes [15, 16]. TCM used for hair loss dates back to the Qin Dynasty two thousand years ago. After thousands of years of development, ancient Chinese medicine has accumulated a large number of effective CHFs and rich experience in treating AGA. In recent years, an increasing number of clinical studies have indicated that the conventional medicines (CM) have been significantly improved when complemented with TCM, and abundant evidence has demonstrated that CHFs with various types of medicinal ingredients can substantially promote the recovery from AGA and significantly lower adverse event rates and recurrence rates. However, the popularizing rate of TCM therapies beyond China is limited, and there is still a lack of reliable evidence that systematically reviews the clinical efficacy and safety of TCM. Therefore, it is necessary to rigorously conduct a systematic review and meta-analysis to assess the efficacy and safety of TCM for AGA to provide a reference for the clinical and rational usage of drugs and individual treatment in an objective manner.

## 2. Methods

### 2.1. Search Strategy

The protocol has been registered in PROSPERO (ID: CRD42019117139). Available online at http://www.crd.york.ac.uk/PROSPERO/display_record.asp?ID=CRD42019117139. The databases from which we retrieved studies in this review included PubMed, WanFang, EMBASE, Web of Science, Cochrane Library, China National Knowledge Infrastructure (CNKI), Chinese Science and Technology Periodical Database (VIP), and Chinese Biomedical (Sino-Med) through March 2019. Because TCM is mainly used in China, we searched these aforementioned Chinese electronic databases to obtain as many clinical trials as possible. The search languages were restricted to Chinese and English. For the English databases, we used the following search strategies: subject terms plus its entry terms. For the Chinese databases, we used subject terms = (“Zhong Yao”) and title = (“Tuo Fa” or “Ban Tu” or “Diao Fa”). Moreover, we manually searched articles meeting our inclusion criteria from other sources, but not obtained from the above 8 databases. Eligible studies were independently screened out by two reviewers. When a discrepancy occurred between the two investigators, it was resolved by discussion. The detailed search strategy for the aforementioned English databases is shown in Table 1.

### 2.2. Article Inclusion and Data Extraction

The systematic review was conducted on the basis of the Preferred Reporting Items for Systematic Review and Meta-Analyses (PRISMA) Statement. We selected eligible studies based on the following inclusion criteria: (1) the TCM groups were treated with TCM regardless of formulation (lotion, pill, decoction, or capsule); (2) the control groups were treated with placebo or conventional therapy; and (3) the TER and total symptom score (TSS) were used as the primary outcome by referring to guidelines, consensus views, or the evaluation criteria. The exclusion criteria were as follows: (1) non-RCTs; (2) the TCM groups received the combination treatment of TCM and acupuncture therapy, or the TCM groups were treated with simple plant extracts; (3) studies did not have control groups or control subjects who received TCM treatment including herbal medicine, acupuncture, or acupoint injection therapy; (4) hair loss induced by chemotherapy or non-AGA; and (5) systematic review, important data reports, and case reports. Two reviewers (Yufen Yan and Suqin Xiong) independently extracted the data, including the following contents: first author's last name, year of publication, sample size, age, TCM name, CM name, duration of treatment, dose, main outcomes, and adverse events.

### 2.3. Quality Assessment

Two reviewers (Fengqing Li and Hao Fang) independently evaluated the methodological qualities of the trials according to the Cochrane Manual [17]. The risk of bias consisted of seven items: selection bias, performance bias, detection bias, attrition bias, reporting bias, and other bias. Each item was classified into low bias risk, high bias risk, and unclear bias risk. Disagreements between the reviewers were settled through discussion.

### 2.4. Data Analysis

In this review, the statistical analysis was conducted by Reviewer Manager (version 5.3.5), and we used OR with 95% CI to analyze dichotomous data, whereas the continuous data were presented as MD or SMD with 95% CI. The data were merged according to the Mantel–Haenszel (fixed-effects) model and the DerSimonian and Laird (random-effects) model [18]. Heterogeneity between the studies was determined by the chi-square test. With the *I*^2^ statistic, an *I*^2^ < 25% indicated that heterogeneity may not be important, values between 25% and 50% represented a moderate inconsistency, and *I*^2^ > 50% suggested severe heterogeneity. We defined *P* ≥ 0.1 and *I*^2^ < 50 as indication that the results have good agreement, and the fixed-effects model would be used, while *I*^2^ > 50% was an indicator of significant heterogeneity among trials. Then, we used a random-effects model to estimate the pooled results to minimize the influence of potential clinical heterogeneity. All the statistical tests were two-tailed, and the differences were statistically significant at *P* < 0.05. Sensitivity analyses were performed to evaluate the robustness of the merged results by removing individual studies. Egger's test was used to evaluate publication bias.

## 3. Results

### 3.1. Search Results and Study Characteristics

**Figure 1:**
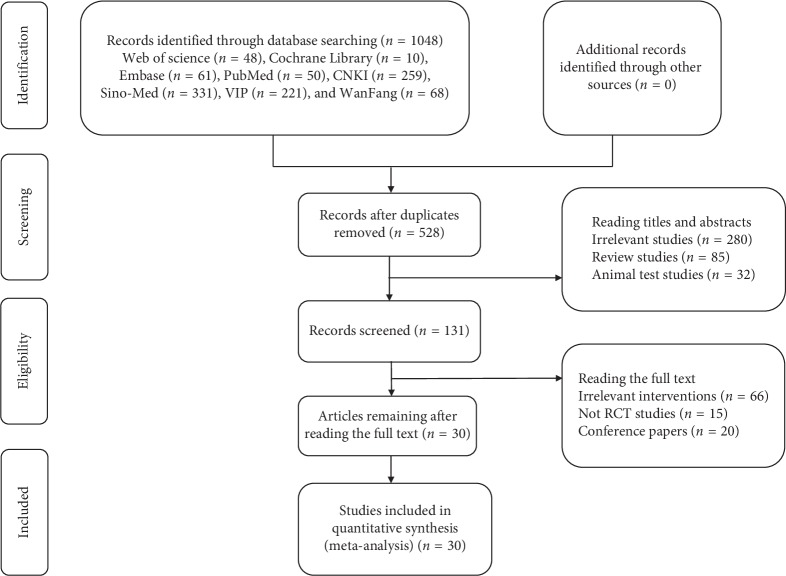
Flowchart of study selection.

In total, 1048 papers were obtained from a database search (Web of Science (*n* = 48), Cochrane Library (*n* = 10), EMBASE (*n* = 61), PubMed (*n* = 50), CNKI (*n* = 259), Sino-Med (*n* = 331), VIP (*n* = 221), and WanFang (*n* = 68)), of which 520 duplicated publications were removed. A total 397 citations of irrelevant topics were excluded after reading the titles and abstracts (irrelevant studies (*n* = 280), review studies (*n* = 85), animal test studies (*n* = 32)), and 101 other studies were ruled out following a screening of the full text (irrelevant interventions (*n* = 66), non-RCT studies (*n* = 15), and conference papers (*n* = 20)). Finally, according to the inclusion criteria, 30 RCTs published between 2008 and 2018 involving 2615 AGA patients were eligible. The sample size was 40 to 185, with a significant age difference. The dosage forms of the TCM group comprised lotion (*n* = 3), decoction (*n* = 25), capsule (*n* = 1), and pill (*n* = 1), and the interventions of the control groups included finasteride, minoxidil, ketoconazole, cystine, vitamins, and selenium disulfide. Eighteen trials reported the specific number of adverse events. Generally, the basic characteristics of the 2615 patients were consistent, and no significant differences were found before the intervention. The literature search process is shown in Figure 1, and the general characteristics of the selected studies are listed in Table 2.

### 3.2. Methodological Quality Assessment

**Figure 2:**
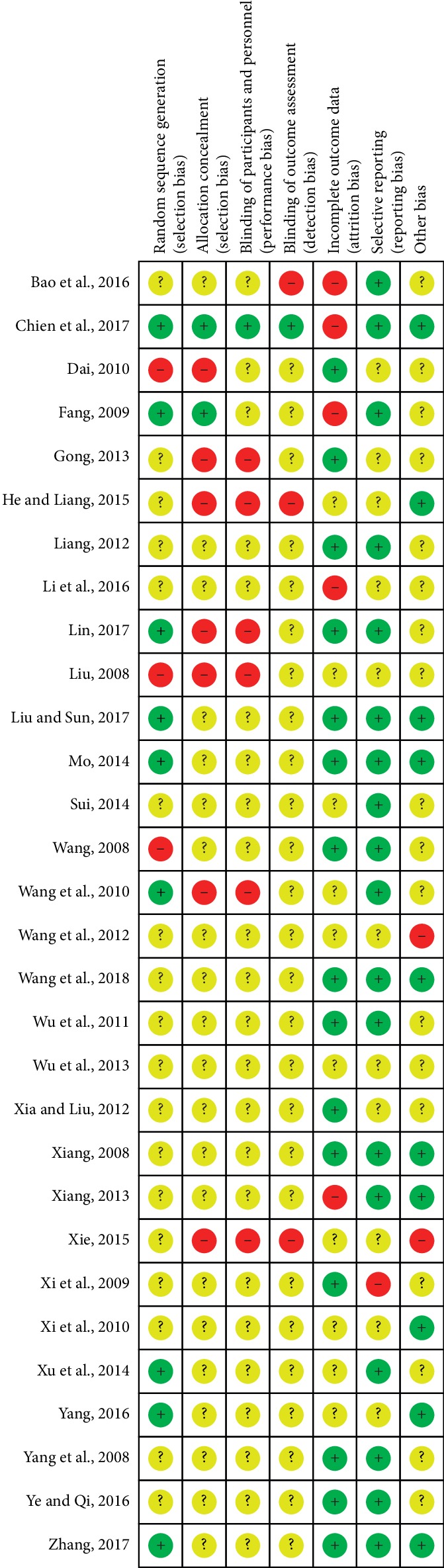
Risk of bias assessment of the 30 articles.

The specific randomized methods were detailed in 9 studies [25, 27, 36–38, 43, 49, 50, 53] which used random number tables or computer or sealed envelopes in the assessment of selection bias. Therefore, we considered them to be low risk. The remaining 21 studies did not offer any detailed information regarding the generation of random sequences. Almost all the studies failed to give the specific allocation concealment, performance bias, or detection bias. Attrition bias was at high risk in four documents [25, 39, 41, 50] due to the number of dropouts and failure to follow up the process of treatment. In general, the quality of the 30 studies was low or remained indistinct because of the high ratio of the unclear risk of biases in most of the studies. The particular results of bias assessment are summarized in Figure 2.

### 3.3. Primary Outcome

#### 3.3.1. Total Efficacy Rate (TER)

**Figure 3:**
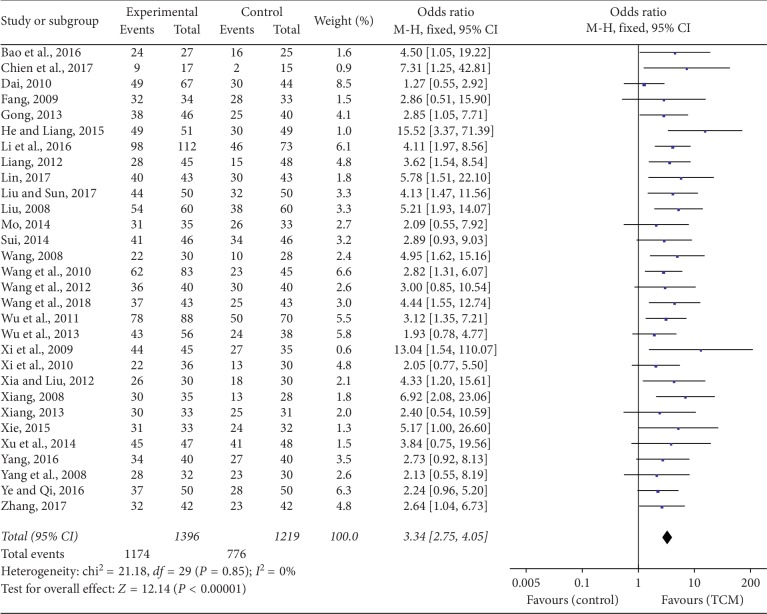
Forest plot and meta-analysis of the TER.

All the studies reported the TER involving 1396 patients in the TCM group and 1219 patients in the CM group. The heterogeneity test (*P*=0.85, *I*^2^ = 0%) suggested that a fixed-effects model was more suitable. The results showed a significant difference between the TCM and CM groups, which indicated that patients in the TCM group benefitted more than those in the CM group (OR = 3.34, 95% CI = 2.75–4.05, *P* < 0.00001), and no difference was found between the subgroups (*P*=0.52, *I*^2^ = 0%), as shown in Figure 3.

#### 3.3.2. TSS

**Figure 4:**
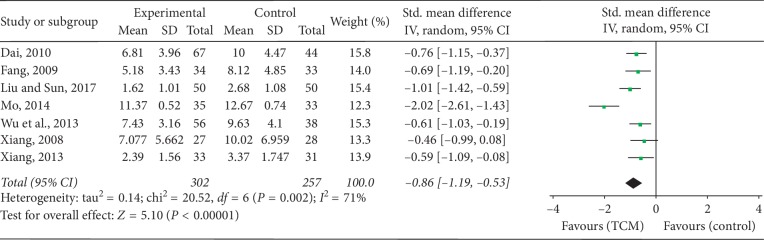
Forest plot and meta-analysis of the TSS.

Seven studies [29, 30, 36, 38, 41, 42, 50] reported the TSS. Because of significant heterogeneity, a standardized mean difference (SMD) with a random-effects model was employed to synthesize the data (*P*=0.002, *I*^2^ = 71%). The results suggested that the TSS of the TCM group was reduced more effectively than that of the CM group (SMD = −0.86; 95% CI = −1.19, −0.53; *P* < 0.00001), as shown in Figure 4.

### 3.4. Secondary Outcome

#### 3.4.1. Common Symptoms

**Figure 5:**
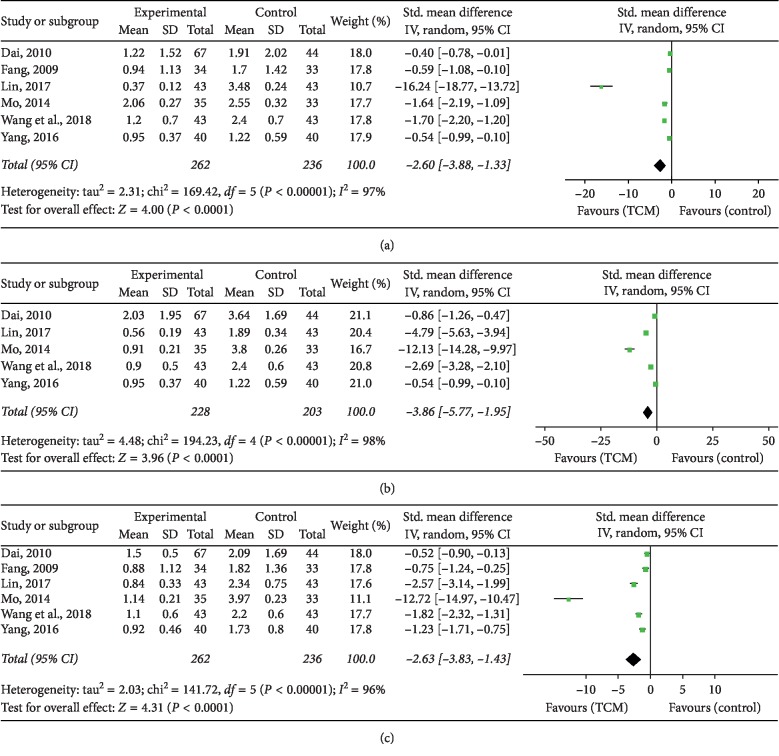
Forest plot and meta-analysis of common symptoms including (a) itchy scalp level, (b) greasy scalp level, and (c) dandruff level.

Several studies reported the common symptoms comprising itchy scalp (*n* = 6), greasy scalp (*n* = 5), and dandruff (*n* = 6). The results suggested that the addition of TCM significantly improved the itchy scalp level (SMD = −2.60, 95% CI = −3.38, −1.33, *P* < 0.0001), greasy scalp level (SMD = −3.86, 95% CI = −5.77, −1.95, *P* < 0.0001), and dandruff level (SMD = −2.63, 95% CI = −3.83, −1.43, *P* < 0.0001) when compared with the control groups, as shown in Figure 5.

#### 3.4.2. Microelement Levels (Ca^2+^, Fe^2+^, Zn^2+^, and Cu^2+^)

**Figure 6:**
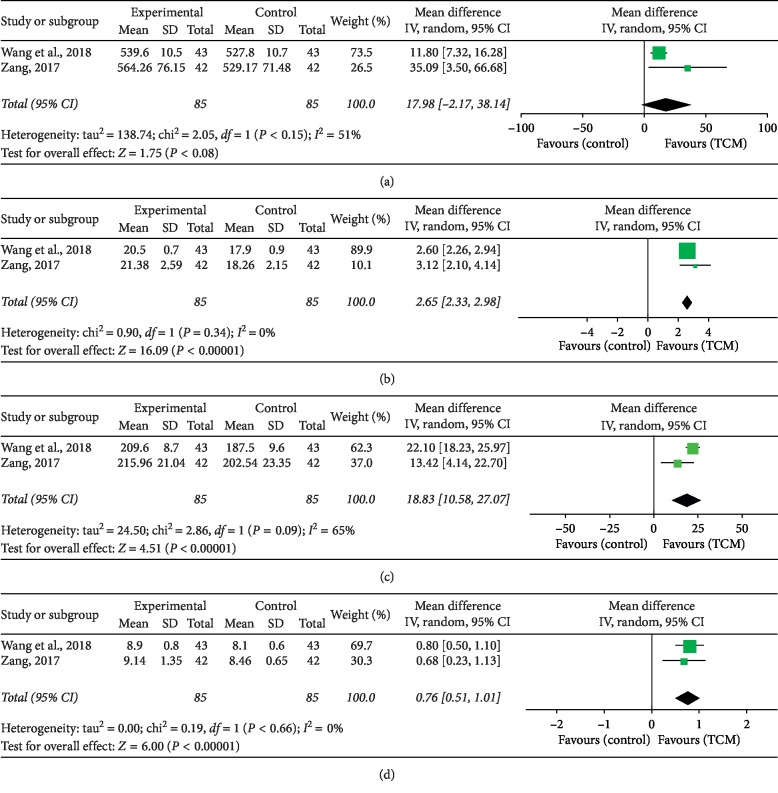
Forest plot and meta-analysis of microelement levels including (a) Ca^2+^, (b) Fe^2+^, (c) Zn^2+^, and (d) Cu^2+^.

The microelement levels of hair including Ca^2+^, Fe^2+^, Zn^2+^, and Cu^2+^ were determined in two trials [34, 37]. The results indicated that in comparison with the CM, the addition of TCM therapy strikingly improved the Fe^2+^ (MD = 2.65, 95% CI = 2.63, 2.98, *P* < 0.00001), Zn^2+^ (MD = 18.89, 95% CI = 10.68, 27.10, *P* < 0.00001), and Cu^2+^ levels (MD = 0.76, 95% CI = 0.51, 1.01, *P* < 0.00001). However, no statistic difference was observed between the TCM and CM groups in terms of the Ca^2+^ level (MD = 17.98, 95% CI = −2.17, 38.14, *P*=0.008), as shown in Figure 6.

### 3.5. Adverse Events

**Figure 7:**
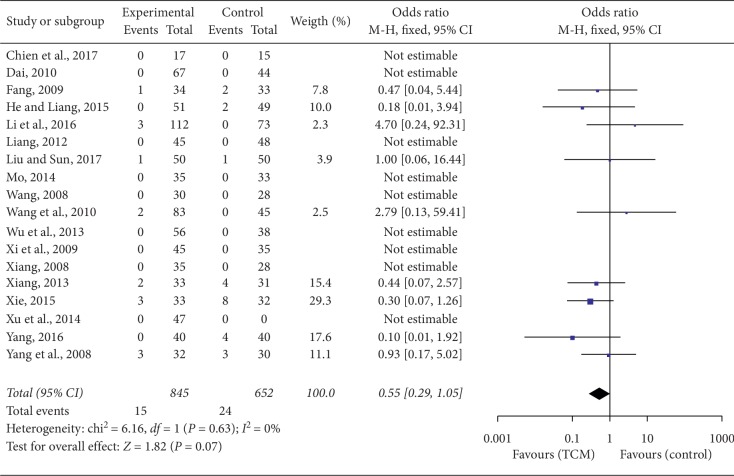
Forest plot and meta-analysis of adverse events between the TCM and control groups.

Eighteen RCTs reported the specific number of adverse events involving 39 patients in the two groups (15 cases for the TCM groups and 24 cases for the control groups). Nine of the 18 studies reported adverse events in their trial [24, 27, 33, 35, 38, 41, 49–51]. The main adverse reactions were dermatitis (pruritus and epifolliculitis) and slight gastrointestinal upset including nausea, vomiting, inappetence, and diarrhea. No serious adverse events (such as liver injury and kidney damage) were mentioned in any of the studies. One study [27] reported that four patients in the control group experienced hypaphrodisia after taking finasteride (4/59, 10%) and were relieved after drug withdrawal. No adverse reactions occurred in another nine trials during the course of treatment [8, 25, 26, 29, 30, 36, 40, 42, 45]. However, we failed to report the adverse events in the remaining 12 studies due to the absence of complete data. This meta-analysis indicated that no heterogeneity was found (*P*=0.63, *I*^2^ = 0%), and there was no significant difference between the TCM and the CM groups in terms of the adverse events (OR = 0.55, 95% CI = 0.29–1.05, *P*=0.07). Although no statistical significance was observed, there was an obvious trend of a decreased total adverse events rate in the TCM group (15/845, 1.8%) when compared with the CM group (24/652, 3.7%). Therefore, the addition of TCM, to some extent, reduced the total adverse events rate, as shown in Figure 7.

### 3.6. Sensitivity Analysis

**Figure 8:**
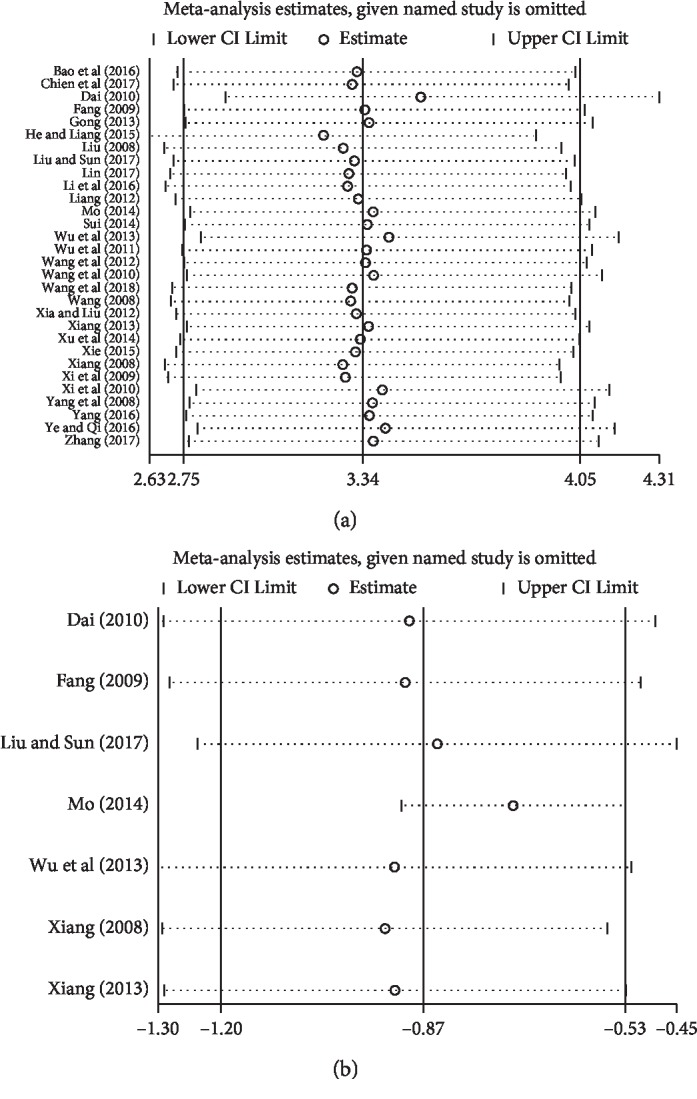
Sensitivity analysis plot of (a) TER and (b) TSS.

Stata 15.1 was applied to the sensitivity analysis of the main outcomes comprising TER and TSS. The results suggested that removing any one study of each outcome had no significant effect on the overall results, indicating that the results of this meta-analysis were reliable, as shown in Figure 8.

### 3.7. Publication Bias

**Figure 9:**
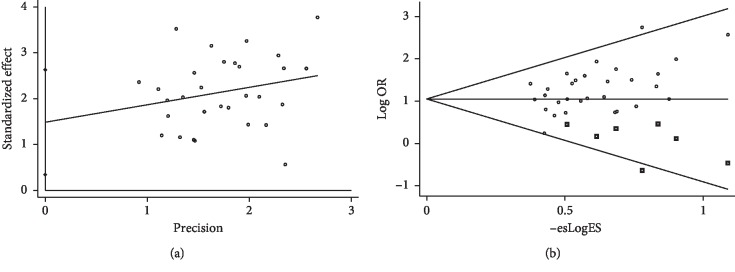
(a) Egger's publication bias and (b) filled funnel plot of TER.

Stata15.1 software was used to detect the possible publication bias of the primary outcome, and the trim and filling method was conducted to cope with striking publication bias if the *P* < 0.05. The result of Egger's test suggested that significant publication bias was observed in terms of TER (*P* > |*t*| = 0.013, 95% CI = 0.34 to 2.6). Then, trim and filling methods were employed to evaluate the reliability of the results affected by significant publication bias [54]. After running the iteration, seven studies marked with squares were filled into the funnel plot. However, the OR and 95% CI after the trim and filling method (OR = 2.86; 95% CI = 2.37–3.44) were consistent with the previous result (OR = 3.34, 95% CI = 2.75–4.05), indicating that the result was stable without the flip, as shown in Figure 9.

## 4. Discussion

AGA is progressive hair loss in the frontotemporal region and top of the head after puberty, which seriously affects the appearance and brings great mental pressure and a psychological burden to patients, especially young people. The incidence rate of AGA across the world is on the rise, with 30.2% in the Asians and higher in Caucasians [52]. According to previous research, the main cause of AGA is genetic factors related to autosomal dominant polygenic inheritance and endocrine dysplasia; insomnia, mental pressure, mental disorders, and inappropriate diet play an important role in aggravating AGA symptoms [47]. Currently, finasteride and minoxidil remain the first-line drugs for AGA. Because a large number of randomized placebo-controlled clinical trials have shown that finasteride and minoxidil significantly improved the hair density, hair diameter, global photographic assessment (*P* < 0.05), and plasma dihydrotestosterone levels [55–58], their curative effects are indisputable. However, because of side effects, a high recurrence rate, and persistent metabolic abnormalities of androgen-induced long-term medication, the efficacy and safety of finasteride and minoxidil need further improvement. Additionally, AGA usually results in depression and anxiety which immensely reduces the efficacy of finasteride and minoxidil. Therefore, finding effective complementary therapies that significantly enhance the effectiveness and lower the adverse events rate of finasteride and minoxidil is a crucial issue for medical investigators worldwide.

Chinese classic herbal formulas documented in the ancient Chinese medical literature have been widely used for AGA for thousands of years. Examples include “Yellow Emperor's Inner Classic” during the Warring States Period (457–221 BC) [59], “Treatise on Febrile Diseases” during the Three Kingdoms period (219 AD), and “Compendium of Materia Medica” during the Ming Dynasty (1552–1578 AD) [38]. With long-term clinical experience, the ancient TCM physicians found that the aetiological agent of AGA were mainly resulted from blood-heat with dry wind, spleen-stomach with dampness-heat, and liver and kidney deficiency [38], and these physicians obtained extensive effective CHFs for the treatment of AGA. Compared with the single therapeutic approach of CM, TCM lays more emphasis on the integrality and multitarget effects of therapy. In recent years, TCM therapies have played a significant role in modern comprehensive treatment. An increasing number of studies have confirmed its unique effect and role in compensating for the deficiencies of CM. Both the decoction and lotion treatment of TCM have exhibited satisfactory effects in easing itchy scalp, greasy scalp, dandruff, and hair loss [27, 29, 34, 36, 43, 50] through the promotion of blood circulation, removing blood stasis [31, 39, 60], tonifying kidney and liver [33, 43], clearing heat and removing toxicity [35], calming the heart and tranquilizing the mind [33], increasing the microelement levels of the hair [34, 37], and downregulating the serum testosterone level [25, 34, 42]. A 2014 meta-analysis [61] was undertaken to assess the efficacy and safety of TCM for AGA, and this meta-analysis concluded that the efficacy of CM therapies was significantly enhanced when complemented with TCM. Despite this positive finding, the conclusion was not convincing because only two outcomes were applied in the review. Moreover, because only 2 studies reported adverse events, the safety assessment of TCM was restricted. Therefore, we rigorously performed an updated systematic review to evaluate the effectiveness and safety of TCM.

This updated meta-analysis assessed the evidence from 30 RCTs with a total 2615 AGA patients randomized to receive additional TCM or CM between 2008 and 2019. The main results included the following: (1) the TER of the TCM group was strikingly higher than that of the CM group (OR = 3.34, 95% CI = 2.75–4.05, *P* < 0.00001), (2) the result of the TSS significantly favored the TCM group when compared with the CM group (MD = –1.29; 95% CI = −1.51, −1.06, *P* < 0.00001), (3) for common symptoms, patients in the TCM group benefited more than those in the CM group, (4) compared with patients treated with the same CM, the addition of TCM provoked a striking improvement in the microelement level (Fe^2+^, Zn^2+^, and Cu^2+^), (5) the results across various subgroups were in great agreement, the benefits of TCM were significant, and no statistical difference was found between the TCM and CM groups regarding the adverse events (OR = 0.55, 95% CI = 0.29–1.05, *P*=0.07).

### 4.1. Primary Outcome

In this meta-analysis, we included as many RCTs as possible to value the clinical effectiveness and safety of TCM therapies to acquire believable evidence for treating AGA. The results indicated that additional TCM significantly improved the TER by 21% when compared with the CM group alone. Regarding the TSS (*I*^2^ = 71%), there was significant heterogeneity in the pooled data. The results of the sensitivity analysis suggested that one study [36] greatly reduced the heterogeneity from 71% to 0. By comparing it with the other 6 trials, we found that it used different scales for symptom scores, which directly led to high heterogeneity. However, we selected this research because it met our inclusion criteria. Generally, the result showed that TCM strikingly reduced the TSS by improving the itchy, greasy, and dandruff levels.

### 4.2. Secondary Outcomes

The secondary outcomes comprised common symptoms and microelement levels (Ca^2+^, Fe^2+^, Zn^2+^, and Cu^2+^). Common symptoms were reported in six relatively high-quality papers [27, 29, 34, 43, 50]. By analyzing the common symptoms, we found that the CHFs used in the six studies contained almost all kidney yang-tonifying herbs (Rehmanniae Radix) and blood-activating herbs (Salviae Miltiorrhizae Radix), which are conventional herbs used to enhance immunity and promote blood circulation for thousands of years in China. Two studies [34, 37] reported that the microelement levels (Fe^2+^, Zn^2+^, and Cu^2+^) of the TCM group were significantly improved when compared with the CM group, indicating that TCM played an important role in nourishing hair by improving some microelement levels. Additionally, the results of two studies [34, 42] showed that the serum testosterone level was greatly downregulated when complemented with TCM containing Salviae Miltiorrhizae Radix. Moreover, animal studies suggested that Salviae Miltiorrhizae Radix significantly lowered the androgen level by inhibiting the expression of SF-1 protein in testicular tissue [62] and steroid hormone synthesis [63, 64]. The possible mechanisms of CHFs in the treatment of AGA were the promotion of hair growth by nourishing hair follicle cells and prevention of epilation by consolidating the root of hair. Because most studies lack cellular and molecular parameters such as hormones, microelements, and biochemical factors, at the cellular and molecular levels, the anti-AGA mechanism of TCM warrants further investigation. In brief, it is likely that TCM through a multiple-target pathway, multilevel and holistic therapy, has shown definite benefits in the adjunctive treatment of AGA.

### 4.3. Subgroup Analysis

A valid subgroup analysis could improve the reliability of these results. In this meta-analysis, a subgroup analysis was performed for the two primary outcomes including the TER and TSS, to identify the difference between the subgroups. In terms of the TER, 22 studies were selected and divided into five subgroups, including TCM versus placebo, TCM versus finasteride, TCM versus minoxidil, TCM + minoxidil versus minoxidil, and TCM versus vitamin B6+ cystine. Since being approved by the FDA in 1992, finasteride, as an effective inhibitor of type II 5*α*R, has been widely used for AGA [7]. It was reported that finasteride could decrease the androgen level of hair follicle cells [65]. Minoxidil, another conventionally used drug, may work by dilating blood vessels, improving the microcirculation around the follicle, increasing skin blood flow, promoting cell division, and prolonging hair follicle growth [66]. However, the pharmacological mechanism of vitamin B6 and cystine in treating hair loss was attributed to the nourishment of follicle cells [67]. The result indicated that additional TCM were more effective than CM alone. With the TSS, two subgroups were introduced to determine the difference between TCM alone and TCM combined therapy. No significant difference was found between the two subgroups (*P*=0.66). Generally, the results among subgroups were inconsistent, as shown in Table 3.

### 4.4. CHFs

There were 29 CHFs used for the treatment of AGA in this review. Two trials used the same CHF, but with different dosage forms (decoction in Xi et al. 2009 and pill in Xi et al. 2010). Although the CHFs used for AGA in most of the included documents were varied, some herbs such as Salviae Miltiorrhizae Radix and Rehmanniae Radix were frequently prescribed in the 29 CHFs. According to the statistical analysis of all ingredients used in the 29 CHFs, we determined the top fifteen frequently used herbs and their usage frequency and listed their efficacies in TCM theory. The results showed that the top fifteen commonly used herbs were Salviae Miltiorrhizae Radix (*n* = 18), Rehmanniae Radix (*n* = 17), Poria (*n* = 17), Polygoni multiflori Radix (*n* = 15), Ligustri Lucidi Fructus (*n* = 15), Ecliptae Herba (*n* = 13), Crataegi Fructus (*n* = 12), Angelicae Sinensis Radix (*n* = 12), Glycyrrhizae Radix et Rhizoma (*n* = 12), Platycladi Cacumen (*n* = 11), Alismatis Rhizoma (*n* = 9), Chuanxiong Rhizoma (*n* = 9), Mori Fructus (*n* = 8), Cuscutae Semen (*n* = 8), and Moutan Cortex (*n* = 8), and the fifteen herbs in bold represent a large proportion (184/333, 55.3%). Although thousands of medicinal herbs are included in the “Chinese pharmacopoeia,” TCM for the treatment of androgenetic alopecia is highly dependent on these 15 herbs. In addition, Rehmanniae Radix, Polygoni multiflori Radix, Ecliptae Herba, Platycladi Cacumen, and Ligustri Lucidi Fructus have been specifically used for hair problems by TCM physicians for thousands of years and are widely recorded in Chinese medicine classic texts. Modern medicine studies the workings of the human body by precisely measuring its cellular, protein, molecular, receptor, target, genetic, and other parameters and emphasizes the treatment of diseases, while TCM pays more attention to the integrity of the human body and emphasizes comprehensive conditioning and personalized medicine. Although they use different strategies, their ultimate goal is the same. Thus, there are some differences between formulations. However, the statistical result suggested they use most of the same herbs. In brief, the 29 CHFs prescribed for treating AGA, based on the basic theory of TCM, were generally consistent, which further explains the prescription regularity of TCM for AGA and provides, to some extent, a theoretical basis for the research and development of new CHFs for the adjuvant treatment of AGA, as shown in Tables 4 and 5.

### 4.5. Limitations and Critical Considerations

Several limitations of our meta-analysis should be highlighted. First, because we only searched the main English and Chinese databases, some studies meeting our inclusion criteria published in other languages or databases may be excluded. All included trials declared that they employed randomization, but only nine studies described a specific randomization method. Additionally, the detailed blinded assessments were not reported in most documents except two studies (Chien et al. 2017; Fang. 2009), which may have exerted a potential impact on the objectivity of the AGA outcomes. Second, most studies had small sample sizes with relatively low-quality designs, which may have led to overvaluing the benefit of TCM. In addition, the composition, dosage form, and treatment duration of the TCM groups varied, which led to significant heterogeneity among the studies. Third, almost half of the studies had only a few endpoint indicators, and many outcomes except TER and TSS were less than six studies. In addition, the evaluation of the therapeutic effect was somewhat subjective by using common symptoms. Fourth, a certain publication bias existed in the 30 documents. However, although all these deficiencies may undermine the quality of evidence of this meta-analysis, the included trials were highly comparable, and we strictly applied the inclusion criteria and followed the guidelines. Since the patients of selected studies were mainly from China, the conclusion of this meta-analysis may not be applicable to other ethnic groups. Therefore, large sample trials with high-quality and well-designed ethnic groupings should be conducted in the future to provide more credible evidence on the efficacy and safety of TCM for AGA.

## 5. Conclusions

This meta-analysis suggested that the TER, clinical symptoms, serum testosterone, and microelement levels of the CM groups were significantly improved when complemented with TCM. Therefore, TCM could be recommended as an effective and safe complementary therapy for the treatment of AGA. However, long-term and higher-quality RCTs are needed to overcome the limitations of the selected studies and more precisely interrogate the effectiveness and safety of TCM.

## Figures and Tables

**Table 1:** Search strategy of the English databases.

Database	Search term A	Search term B	Strategy
PubMed	[Title/abstract] = (traditional Chinese medicine OR Zhong Yi Xue OR Zhong yao OR herbal medicine OR phytotherapy OR Chinese medicine OR phytotherapies OR Chinese herbal compound OR Chinese herbal formula^*∗*^ OR Chinese compound formula^*∗*^ OR TCM)	[Title/abstract] = (alopecia OR Baldness^*∗*^ OR hair Loss^*∗*^ OR calvities OR balding OR AGA)	Term A and Term B

EMBASE	[Title/abstract] = (traditional Chinese medicine OR Zhong Yi Xue OR Zhong yao OR herbal medicine OR phytotherapy OR Chinese medicine OR phytotherapies OR Chinese herbal compound OR Chinese herbal formula^*∗*^ OR Chinese compound formula^*∗*^ OR TCM)	[Title/abstract] = (alopecia OR Baldness^*∗*^ OR hair Loss^*∗*^ OR calvities OR balding OR AGA)	Term A and Term B

Web of Science	TS = (“traditional Chinese medicine” or “herbal medicine” or “Zhong Yi Xue” or “Zhong Yao” or “phytotherapy” or “Chinese medicine” or “phytotherapies” or “Chinese herbal compound^*∗*^” or “Chinese herbal formula^*∗*^” or “Chinese compound formula^*∗*^” or “TCM”)	TS = (“alopecia^*∗*^” or “baldness^*∗*^” or “calvities” or “Hair Loss^*∗*^” or “balding^*∗*^” or “AGA”)	Term A and Term B

Cochrane library	[Title/abstract] = (traditional Chinese medicine OR Zhong Yi Xue OR Zhong yao OR herbal medicine OR phytotherapy OR Chinese medicine OR phytotherapies OR Chinese herbal compound OR Chinese herbal formula^*∗*^ OR Chinese compound formula^*∗*^ OR TCM)	[Title] = (alopecia OR baldness^*∗*^ OR hair Loss^*∗*^ OR calvities OR balding OR AGA)	Term A and Term B

**Table 2:** The general characteristics of the 30 trials.

Study	Sample size (T/C)	Age (mean or range) T/C	Diagnostic criteria/classification [19–23]	Intervention		Duration of use (months)	Adverse reactions		Main outcome
				T	C		T	C	
Yang et al. [24]	32/30	20–40	Guiding principles for clinical research of new Chinese medicine	ShengFa Lotion (5 ml, qod, local)	Placebo (5 ml, qod local)	12	3	3	①
Chien et al. [25]	20/20	39.85 ± 8.77/35.30 ± 7.19	Hamilton–Norwood	BeauTop (2.4 g, bid, po)	Placebo (2.4 g, bid, po)	6	0	0	①
Liang et al. [26]	45/48	21.3 ± 1.58/21.1 ± 1.04	Clinical dermatology	YuFaYe Lotion (4 ml, bid, local)	Placebo (4 ml, bid, local)	4	0	0	①
Yang [27]	40/40	28.4 ± 6.2/28.7 ± 6.0	Hamilton–Norwood (II-VI)	YiRen QuShi Decoction (1 dose, qd, po)	Finasteride (1 mg, qd, po)	3	0	4	①②③④
Wu et al. [28]	84/74	29.34 ± 7.13/28.41 ± 6.64	Clinical dermatology	TuoFaFang Decoction (150 ml, bid, po)	Finasteride (1 mg, qd, po)	3	NR	NR	①
Dai [29]	67/44	20–45	Clinical dermatology Hamilton–Norwood (II-VI)	QuShi ShengFaFang Decoction (1 dose, qd, po)	Finasteride (1 mg, qd, po)	3	0	0	①②③④
Wu et al. [30]	58/36	27.8 ± 5.9/28.47 ± 5.13	Clinical dermatology Hamilton–Norwood (II-VI)	QuShi ShengFa Decoction (1 dose, qd, po)	Finasteride (1 mg, qd, po)	6	0	0	①⑤
Sui [31]	46/46	26.43 ± 7.01/26.54 ± 6.62	Clinical dermatology	QuZhi GuTuoYin Decoction (200 ml, bid, po)	Finasteride (1 mg, qd, po)	3	NR	NR	①
Wang et al. [32]	40/40	27.32 ± 6.06/27.14 ± 5.58	Clinical diagnosis and treatment of TCM dermatology	QuShi JianFaYin Decoction (200 ml, bid, po)	Finasteride (1 mg, qd, po)	6	NR	NR	①
Li et al. [33]	112/73	26.42 ± 5.63/25.87 ± 6.12	Chinese guidelines for the diagnosis and treatment of androgenetic alopecia	ShengFa Pill Decoction (1 dose, qd, po)	Finasteride (1 mg, qd, po)	6	3	0	①
Wang and Zhang [34]	43/43	29.7 ± 8.5/28.6 ± 8.3	Etiology, diagnosis, and treatment of androgenetic alopecia	QuZhi ShengFaYin Decoction (150 ml, bid, po) Finasteride (1 mg, qd, po)	Finasteride (1 mg, qd, po)	6	NR	NR	①②③④⑥
He and Liang [35]	51/49	29.54 ± 5.79/29.92 ± 6.43	Hamilton (II–V)	DiHuang ShengFaLing Decoction (5 g, bid, po)	2%–5% minoxidil (1 ml, bid, local)	9	0	2	①
Mo [36]	35/33	18–50	Clinical dermatology	QuZhi ShengFa Lotion (50 ml, qid, local)	5% minoxidil (1 ml, bid, local)	6	0	0	①②③④
Zhang [37]	42/42	20–32	Clinical dermatology	JiaWei ErZhiWan Decoction (150 ml, bid, po) 5% minoxidil (1 ml, bid, local)	5% minoxidil (1 ml, bid, local)	6	NR	NR	①⑥
Liu and sun [38]	50/50	28.35 ± 4.52/28.23 ± 4.35	Clinical dermatology Hamilton (II–V)	YiShen ShengFaTang Decoction (150 ml, bid, po) 5% minoxidil (1 ml, bid, local)	5% minoxidil (1 ml, bid, local)	3	1	1	①
Bao et al. [39]	29/28	27.96 ± 10.5/29.18 ± 9.787	MAGA (I-III) Ludwig (I-III)	Gao Fang Decoction (20 ml, bid, po) 5% minoxidil (1 ml, bid, local)	5% minoxidil (1 ml, bid, local)	4	NR	NR	①
Wang [40]	30/28	35.27 ± 5.74/35.57 ± 5.98	Clinical dermatology Hamilton–Norwood (II-VI)	BuShen ZiYing Fang Decoction (1 dose, qd, po) Finasteride (1 mg, qd, po) 5% minoxidil (1 ml, bid, local)	Finasteride (1 mg, qd, po) 5% minoxidil (1 ml, bid, local)	6	0	0	①②③④
Xiang [41]	40/40	18–50	Hamilton–Norwood (II-VI)	ZhaQu PinWeiSan He Er ZhiWan Decoction (150 ml, tid, po) 5% minoxidil (1 ml, bid, local)	Finasteride (1 mg, qd, po) 5% minoxidil (1 ml, bid, local)	9	2	4	①⑤
Xiang [42]	35/28	28.3 ± 6.7/30.5 ± 5.3	Clinical dermatology, Norwood-Ludwig	Tanshinone Capsule (2#, tid, po)	VitaminB2 (5 mg, tid, po) Vitamin B6 (10 mg, tid, po)	3	0	0	①⑤
Lin [43]	43/43	59.44 ± 2.57/58.16 ± 2.98	Clinical dermatology	Qibao beards folk prescription Decoction (200 ml, bid, po)	VitaminB6 (10 mg, tid, po) Cystine (50 mg, tid, po)	4	NR	NR	①②③④
Gong [44]	46/46	20–48	Integrated Chinese and western medicine dermatology	JiaWei HuangLian EJiao Decoction (200 ml, bid, po)	VitaminB6 (10 mg, tid, po) Cystine (50 mg, tid, po)	3	NR	NR	①
Xi et al. [45]	45/35	30.1 ± 12.8/29.7 ± 11.6	Clinical dermatology	GuShen ShengFaTang Decoction (150 ml, bid, po)	VitaminB6 (10 mg, tid, po) Cystine (50 mg, tid, po)	3	0	0	①
Xia and Liu [46]	30/30	25–51	Clinical dermatology	ZiNi ZiShen YangXue ShengFaTang Decoction (300 ml, bid, po) 5% minoxidil (1 ml, bid, local)	VitaminB6 (10 mg, tid, po) Cystine (50 mg, tid, po) 5% minoxidil (1 ml, bid, local)	6	NR	NR	①
Ye and Qi [47]	25/25	17–50	Clinical diagnosis and treatment of TCM dermatology	YangXue ShengFa HeJi Decoction (30 ml, tid, po)	VitaminB6 (10 mg, tid, po) Cystine (50 mg, tid, po)	3	NR	NR	①
Xi et al. [48]	36/30	29.3 ± 4.7/29.6 ± 4.2	Hamilton (II-IV)	QuZhi ShengFa Pill (9 g, tid, po)	VitaminB6 (10 mg, tid, po) Cystine (50 mg, tid, po)	3	NR	NR	①
Wang et al. [49]	83/45	26/29	Clinical diagnosis and treatment of TCM dermatology	Fukang mixture Decoction (20 ml, tid, po)	VitaminB6 (20 mg, tid, po) Cystine (10 mg, tid, po)	4	2	0	①
Fang [50]	36/36	30.62 ± 10.04/29.94 ± 9.46	Clinical dermatology	ShengFa Decoction (20 ml, tid, po) 5% minoxidil (1 ml, bid, local)	VitaminB6 (10 mg, tid, po) Cystine (50 mg, tid, po) 5% minoxidil (1 ml, bid, local)	6	1	2	①②④⑤
Xie [51]	33/32	45.5 ± 1.2/45.6 ± 1.4	Guiding principles for clinical research of new Chinese medicine	JianWei YiShen, QuShi ShengFa Decoction (bid, po)	21-Super-Vita (0.7 g, tid, po) Cystine (100 mg, tid, po) 2%KCZ (biw)	3	3	8	①
Liu [52]	60/60	42 ± 7.9/42 ± 7.8	Integrated Chinese and western medicine dermatology	Quzhi Fangtuo shengfa ying Decoction (100 ml, biw, local)	21-Super-Vita (0.7 g, tid, po) Cystine (100 mg, tid, po) 2%KCZ (biw)	3	NR	NR	①
Xu et al. [53]	47/48	32.15 ± 4.62/31.21 ± 4.16	Clinical dermatology	ShengFa Ting Decoction (biw, local) Selenium sulfide (biw)	Selenium sulfide (biw)	3	0	0	①

T: experimental group; C: control group; KCZ: ketoconazole; NR: no record; ①: total efficacy rate; ②: itchy scalp level; ③: greasy scalp level; ④: dandruff level; ⑤: total symptom score; and ⑥: microelement level.

**Table 3:** Subgroup analysis.

Subgroups	Trials	Effects model	Pooled effect	95% CI	*P* value
TER					
TCM versus placebo	3	Fixed	OR 3.55	1.83–6.88	0.0002
TCM versus finasteride	7	Fixed	OR 2.58	1.83–3.64	<0.00001
TCM versus minoxidil	2	Fixed	OR 5.87	2.30–14.96	0.0002
TCM + minoxidil versus minoxidil	3	Fixed	OR 3.46	1.86–6.44	<0.0001
TCM versus vitamin B6+ cystine	7	Fixed	OR 3.26	2.23–4.78	<0.00001
Total 95%	22	Fixed	OR 3.14	2.53–3.90	<0.00001
Test for subgroup differences: chi-square = 3.22. *df* = 4 (*P*=0.52). *I*_2_ = 0%					

TSS					
TCM versus conventional medicine	4	Random	MD-2.07	–3.12, –1.12	0.0001
TCM + conventional medicine versus conventional medicine	3	Random	MD-1.19	–1.8, –0.58	0.0001
Total 95%	7	Random	MD-1.46	–1.91, –1.01	<0.00001
Test for subgroup differences: chi-square = 0.2. *df* = 1 (*P*=0.66). *I*_2_ = 0%					

**Table 4:** The details of the 30 Chinese herbal formulas.

Study	Formula	Ingredients/percentages	Preparation methods
Yang et al. [24]	ShengFa Lotion	**Salviae Miltiorrhizae Radix** (1.7%), **Polygoni multiflori Radix** (1.25%), Sophorae Flavescentis Radix (1.25%), Zanthoxylum bungeanum (0.8%), AES (15%), AS (5%), BS-12(5%), inorganic additive (10%), glycol distearate (3%), silicone oil (1%), flavours (1%), water (53%)	(1) Diacolation with 5000 ml 95% ethanol (2) Concentrated to a thin extract

Chien et al. [25]	BeauTop Tablet	**Rehmanniae Radix**, **Angelicae Sinensis Radix**, **Ecliptae Herba**, Ginseng Radix, Astragali Radix, Ligustri Fructus	Produced by Sun Ten Pharmaceutical (Taipei, China)

Liang [26]	YuFaYe Lotion	**Angelicae Sinensis Radix**, **Chuanxiong Rhizoma**, Zanthoxylum bungeanum Maxim, Carthami Flos, Zingiberis Rhizoma	Produced by Bawang Co. LTD (Guangzhou, China)

Yang [27]	YiRen QuShi Decoction	**Rehmanniae Radix** (15 g), **Salviae Miltiorrhizae Radix** (15 g), **Platycladi Cacumen** (15 g), **Poria** (10 g), **Crataegi Fructus** (15 g), **Alismatis Rhizoma** (10 g), **Moutan Cortex** (10 g), Lycopi Herba (10 g), Acori Tatarinowii Rhizoma (10 g), Artemisiae Scopariae Herba (10 g), Chaenomelis Fructus (10 g), Liuyi powder (10 g), Coicis Semen (10 g)	Decocted with water

Wu et al. [28]	TuoFaFang Decoction	**Angelicae Sinensis Radix** (15 g), **Polygoni multiflori Radix** (15 g), **Platycladi Cacumen** (15 g), **Chuanxiong Rhizoma** (15 g), Cinnamomi Ramulus (15 g), Viticis Fructus (15 g), Puerariae Lobatae Radix (30 g)	Decocted with water

Dai [29]	QuShi ShengFaFang Decoction	**Rehmanniae Radix** (15 g), **Salviae Miltiorrhizae Radix** (15 g), **Chuanxiong Rhizoma** (10 g), **Platycladi Cacumen** (15 g), **Crataegi Fructus** (15 g), **Glycyrrhizae Radix et Rhizoma** (5 g), **Poria** (10 g), **Alismatis Rhizoma** (10 g), **Moutan Cortex** (10 g), Chaenomelis Fructus (10 g), Coicis Semen (15 g), Lycopi Herba (10 g)	Decocted with water

Wu et al. [30]	QuShi ShengFa Decoction	**Rehmanniae Radix** (15 g), **Salviae Miltiorrhizae Radix** (15 g), **Poria** (10 g), **Platycladi Cacumen** (15 g), **Crataegi Fructus** (15 g), **Alismatis Rhizoma** (10 g), **Moutan Cortex** (10 g), Lycopi Herba (10 g), Acori Tatarinowii Rhizoma (10 g), Artemisiae Scopariae Herba (10 g), Chaenomelis Fructus (10 g), Liuyi powder (10 g), Coicis Semen (15 g)	Decocted with water

Sui [31]	QuZhi GuTuoYin Decoction	**Salviae Miltiorrhizae Radix** (20 g), **Polygoni multiflori Radix** (15 g), **Glycyrrhizae Radix et Rhizoma** (6 g), **Crataegi Fructus** (15 g), **Poria** (30 g), **Ligustri Lucidi Fructus** (15 g), **Moutan Cortex** (10 g), **Cuscutae Semen** (12 g), Sophorae Flavescentis Radix (10 g), Astragali Radix (15 g), Polyporus (15 g), Lycii Fructus (10 g), Psoraleae Fructus (12 g), Ziziphi Spinosae Semen (15 g)	Decocted with water

Wang et al. [32]	QuShi JianFaYin Decoction	**Rehmanniae Radix** (20 g), **Chuanxiong Rhizoma** (15 g), **Alismatis Rhizoma** (15 g), **Mori Fructus** (15 g), Polyporus (25 g), Tuber Fleeceflower Stem (25 g), Dioscoreae Tokoro Rhizoma (25 g), Plantaginis Semen (15 g), Dictamni Cortex (25 g), Atractylodis Macrocephalae Rhizoma (25 g), Halloysitum Rubrum (20 g)	Decocted with water

Li et al. [33]	ShengFa Pill Decoction	**Rehmanniae Radix** (12–30 g), **Salviae Miltiorrhizae Radix** (15–30 g), **Angelicae Sinensis Radix** (10 g), **Crataegi Fructus** (10 g), **Chuanxiong Rhizoma** (10 g), **Alismatis Rhizoma** (10 g), **Cuscutae Semen** (12 g), Chaenomelis Fructus (6 g), Ziziphi Spinosae Semen (15–20 g), Paeoniae Radix Alba (12 g), Polygoni Multiflori Caulis (15 g), Carthami Flos (6–10 g), Cimicifugae Rhizoma (3 g), Atractylodis Macrocephalae Rhizoma (10–15 g)	Decocted with water

Wang and Zhang [34]	QuZhi ShengFaYin Decoction	**Salviae Miltiorrhizae Radix** (30 g), **Polygoni multiflori Radix** (15 g), **Poria** (20 g), **Crataegi Fructus** (15 g), **Ligustri Lucidi Fructus** (15 g), **Glycyrrhizae Radix et Rhizoma** (10 g), **Mori Fructus** (15 g), **Alismatis Rhizoma** (15 g), **Moutan Cortex** (15 g), Taraxaci Herba (25 g), Oldenlandia diffusa Herba (30 g), Coicis Semen (40 g), Gardenia jasminoides (20 g), Dictamni Cortex (20 g)	Decocted with water

He and Liang [35]	DiHuang ShengFaLing Decoction	**Rehmanniae Radix** (200 g), **Ligustri Lucidi Fructus** (150 g), **Chuanxiong Rhizoma** (40 g), **Ecliptae Herba** (100 g), Carthami Flos (40 g), Psoraleae Fructus (150 g), Cordyceps (20 g), Ginseng Radix (50 g), Achyranthis bidentate Radix (50 g), Morindae Officinalis Radix (120 g), Polygonati Rhizoma (120 g)	Produced by Jingchuan Hospital of Traditional Chinese Medicine, Gansu, China

Mo [36]	QuZhi ShengFa Lotion	**Platycladi Cacumen**, Garden Balsam Stem, Sophorae Flavescentis Radix, Polygonati Rhizoma, Gleditsiae Fructus, 0.2% sodium benzoate	Produced by Hunan University of Traditional Chinese Medicine, Changsha, China

Zhang [37]	JiaWei ErZhiWan Decoction	**Rehmanniae Radix** (15 g), **Salviae Miltiorrhizae Radix** (20 g), **Polygoni multiflori Radix** (20 g), **Poria** (10 g), **Ligustri Lucidi Fructus** (20 g), **Glycyrrhizae Radix et Rhizoma** (6 g), **Alismatis Rhizoma** (10 g), **Moutan Cortex** (10 g), **Ecliptae Herba** (20 g), Paeoniae Radix Alba (15 g), Sesami Semen Nigrum (15 g), Juglandis Semen (12 g), Corni Fructus (12 g)	Decocted with water

Liu and Sun [38]	YiShen ShengFaTang Decoction	**Rehmanniae Radix** (20 g), **Angelicae Sinensis Radix** (10 g), **Polygoni multiflori Radix** (15 g), **Poria** (10 g), **Ligustri Lucidi Fructus** (20 g), **Platycladi Cacumen** (15 g), **Glycyrrhizae Radix et Rhizoma** (10 g), **Cuscutae Semen** (20 g), **Ecliptae Herba** (15 g), Lycii Fructus (10 g), Polygonati Rhizoma (15 g), Ziziphi Spinosae Semen (30 g)	Decocted with water

Bao et al. [39]	Gao Fang Decoction	**Rehmanniae Radix** (30 g), **Salviae Miltiorrhizae Radix** (15 g), **Angelicae Sinensis Radix** (20 g), **Polygoni multiflori Radix** (20 g), **Poria** (20 g), **Ligustri Lucidi Fructus** (10 g), **Crataegi Fructus** (15 g), **Chuanxiong Rhizoma** (20 g), **Chuanxiong Rhizoma** (20 g), **Mori Fructus** (20 g), **Ecliptae Herba** (10 g), Psoraleae Fructus (20 g), Dioscoreae Rhizoma (15 g), Paeoniae Radix Alba (20 g), Bupleuri Radix (15 g), Gastrodiae Rhizoma (10 g), Uncariae Ramulus Cum Uncis (10 g), Acori Tatarinowii Rhizoma (20 g), Atractylodis Macrocephalae Rhizoma (30 g), Citri Reticulatae Pericarpium (20 g), Massa Medicata Fermentata (15 g)	Decocted with water

Wang [40]	BuShen ZiYing Fang Decoction	**Salviae Miltiorrhizae Radix** (30 g), **Angelicae Sinensis Radix** (15 g), **Polygoni multiflori Radix** (15 g), **Poria** (15 g), **Ligustri Lucidi Fructus** (30 g), **Glycyrrhizae Radix et Rhizoma** (10 g), **Mori Fructus** (15 g), **Cuscutae Semen** (15 g), **Ecliptae Herba** (30 g), Lycii Fructus (10 g), Astragali Radix (15 g), Taxilli Herba (15 g)	Decocted with water

Xiang [41]	ZhaQu PinWeiSan He Er ZhiWan Decoction	**Salviae Miltiorrhizae Radix** (30 g), **Polygoni multiflori Radix** (30 g), **Ligustri Lucidi Fructus** (30 g), **Crataegi Fructus** (30 g), **Glycyrrhizae Radix et Rhizoma** (6 g), **Platycladi Cacumen** (30 g), **Ecliptae Herba** (15 g), Massa Medicata Fermentata (20 g), Sophorae Flos (30 g), Atractylodis Rhizoma (10 g), Magnoliae officinalis Cortex (15 g), Citri Reticulatae Pericarpium (15 g), Acori Tatarinowii Rhizoma (10 g)	Decocted with water

Xiang [42]	Tanshinone Capsule	**Salviae Miltiorrhizae Radix**	Produced by Hebei Xinglong Xili Pharmaceutical Co. LTD, China

Lin [43]	Qibao beards folk prescription Decoction	**Rehmanniae Radix** (12 g), **Salviae Miltiorrhizae Radix** (15 g), **Angelicae Sinensis Radix** (15 g), **Polygoni multiflori Radix** (18 g), **Poria** (12 g), **Platycladi Cacumen** (10 g), **Ligustri Lucidi Fructus** (18 g), **Mori Fructus** (18 g), **Cuscutae Semen** (15 g), Lycii Fructus (10 g), Achyranthis Bidentatae Radix (12 g), Scutellariae Radix (12 g), Astragali Radix (15 g)	Decocted with water

Gong [44]	JiaWei HuangLian EJiao Decoction	**Rehmanniae Radix** (12 g), **Salviae Miltiorrhizae Radix** (12 g), **Polygoni multiflori Radix** (12 g), Coptidis Rhizoma (12 g), Sophorae Flavescentis Radix (12 g), Paeoniae Radix Alba (12 g), Scutellariae Radix (9 g), Gastrodiae Rhizoma (9 g), Asini Corii Colla (10 g), Hen egg yolk (2#)	Decocted with water

Xi et al. [45]	GuShen ShengFaTang Decoction	**Rehmanniae Radix** (20 g), **Salviae Miltiorrhizae Radix** (20 g), **Polygoni multiflori Radix** (15 g), **Platycladi Cacumen** (15 g), **Crataegi Fructus** (20 g), **Poria** (20 g), **Glycyrrhizae Radix et Rhizoma** (6 g), **Ligustri Lucidi Fructus** (20 g), **Mori Fructus** (20 g), **Ecliptae Herba** (15 g), Taraxaci Herba (20 g), Concha ostreae (30 g), Mori Cortex (15 g), Oldenlandia diffusa Herba (15 g), Bupleuri Radix (10 g)	Decocted with water

Xia and Liu [46]	ZiNi ZiShen YangXue ShengFaTang Decoction	**Rehmanniae Radix** (15 g), **Salviae Miltiorrhizae Radix** (20 g), **Angelicae Sinensis Radix** (15 g), **Polygoni multiflori Radix** (15 g), **Poria** (30 g), **Ligustri Lucidi Fructus** (15 g), **Glycyrrhizae Radix et Rhizoma** (20 g), **Chuanxiong Rhizoma** (20 g), **Mori Fructus** (20 g), **Cuscutae Semen** (15 g), **Ecliptae Herba** (15 g), Dioscoreae Rhizoma (15 g), Paeoniae Radix Alba (10 g), Astragali Radix (15 g), Spatholobi Caulis (20 g), Ziziphi Spinosae Semen (20 g), Tuber Fleeceflower Stem (20 g), Corni Fructus (20 g), Lycii Fructus (20 g), Eucommiae Cortex (15 g), Polygonati Rhizoma (15 g), Taraxaci Herba (20 g)	Decocted with water

Ye and Qi [47]	YangXue ShengFa HeJi Decoction	**Angelicae Sinensis Radix**, **Polygoni multiflori Radix**, **Poria**, **Cuscutae Semen**, Lycii Fructus, Achyranthis Bidentatae Radix, Psoraleae Fructus	Produced by Kunming Hospital of Traditional Chinese Medicine

Xi et al. [48]	QuZhi ShengFa Pill	**Rehmanniae Radix**, **Salviae Miltiorrhizae Radix**, **Polygoni multiflori Radix**, **Platycladi Cacumen**, **Crataegi Fructus**, **Poria**, **Glycyrrhizae Radix et Rhizoma**, **Ligustri Lucidi Fructus**, **Mori Fructus**, **Ecliptae Herba**, Taraxaci Herba, Ostreae Concha, Mori Cortex, Oldenlandia diffusa Herba, Bupleuri Radix	Produced by Hunan University of Traditional Chinese Medicine, Changsha, China

Wang et al. [49]	Fukang mixture Decoction	**Poria** (30 g), **Crataegi Fructus** (30 g), **Moutan Cortex** (10 g), Scutellariae Radix (15 g), Platycodonis Radix (6 g), Taraxaci Herba (30 g), Bupleuri Radix (10 g), Oldenlandia diffusa Herba (20 g), Paeoniae Radix Rubra (30 g), Sophorae Flos (10 g), Dictamni Cortex (15 g), Prunellae Spica (10 g), gypsum (30 g)	Decocted with water

Fang [50]	ShengFa Decoction	**Rehmanniae Radix** (20 g), **Angelicae Sinensis Radix** (15 g), **Ligustri Lucidi Fructus** (10 g), **Moutan Cortex** (15 g), **Cuscutae Semen** (20 g), **Ecliptae Herba** (30 g), Scutellariae Radix (15 g), Mori Cortex (15 g), Schizonepetae Herba (15 g), Saposhnikoviae Radix (10 g), Coicis Semen (30 g), Cicadae Periostracum (10 g)	Decocted with water

Xie [51]	JianWei YiShen QuShi ShengFa Decoction	**Rehmanniae Radix** (15 g), **Poria** (15 g), **Glycyrrhizae Radix et Rhizoma** (6 g), **Crataegi Fructus** (10 g), **Ligustri Lucidi Fructus** (15 g), **Alismatis Rhizoma** (10 g), **Ecliptae Herba** (15 g), Atractylodis Macrocephalae Rhizoma (10 g), Artemisiae Scopariae Herba (15 g), Dictamni Cortex (10 g), Coicis Semen (15 g)	Decocted with water

Liu [52]	Quzhi Fangtuo shengfa ying Decoction	**Angelicae Sinensis Radix** (15 g), **Ligustri Lucidi Fructus** (15 g), **Glycyrrhizae Radix et Rhizoma** (6 g), **Poria** (15 g), **Platycladi Cacumen** (10 g), **Chuanxiong Rhizoma** (15 g), **Alismatis Rhizoma** (10 g), **Ecliptae Herba** (15 g), Coicis Semen (15 g), Puerariae Radix (15 g), Tribuli Fructus (15 g), Dioscoreae Tokoro Rhizoma (10 g), Rubi Fructus (15 g)	Decocted with water

Xu et al. [53]	ShengFa Ting Decoction	**Salviae Miltiorrhizae Radix** (60 g), Psoraleae Fructus (60 g), Astragali Radix (60 g), Carthami Flos (40 g), Zingiberis Rhizoma (60 g), Cinnamomum camphora (50 g)	Diacolation with 1000 ml 60% ethanol

Bold values represent the top 15 commonly used herbs, which have been listed in Table 5.

**Table 5:** Frequency of usage and TCM efficacy of the top 15 commonly used herbs.

Herbs	Frequency	TCM efficacy [68]
Salviae Miltiorrhizae Radix	18	Invigorating blood circulation to dissolve stasis
Rehmanniae Radix	17	Nourishing yin and tonifying blood
Poria	17	Inducing diuresis to alleviate edema
Polygoni multiflori Radix	15	Replenish blood and promoting hair growth
Ligustri Lucidi Fructus	15	Blacking hair
Ecliptae Herba	13	Nourishing liver and kidney and blacking hair
Crataegi Fructus	12	Promoting qi and dissipating stasis
Angelicae Sinensis Radix	12	Tonifying and activating blood
Glycyrrhizae Radix et Rhizoma	12	Tonifying spleen and replenishing qi
Platycladi Cacumen	11	Promoting hair growth and blacking
Alismatis Rhizoma	9	Dampness-draining diuretic
Chuanxiong Rhizoma	9	Activating blood and promoting qi
Mori Fructus	8	Nourishing yin and tonifying blood
Cuscutae Semen	8	Nourishing liver and kidney
Moutan Cortex	8	Activating blood and dissolving stasis
Total	184	
